# Estradiol Modulates Translocator Protein (TSPO) and Steroid Acute Regulatory Protein (StAR) via Protein Kinase A (PKA) Signaling in Hypothalamic Astrocytes

**DOI:** 10.1210/en.2013-1844

**Published:** 2014-05-30

**Authors:** Claire Chen, John Kuo, Angela Wong, Paul Micevych

**Affiliations:** Departments of Obstetrics/Gynecology (C.C., J.K.) and Neurobiology (A.W., P.M.), David Geffen School of Medicine at UCLA, and Laboratory of Neuroendocrinology of the Brain Research Institute (A.W., P.M.), University of California, Los Angeles, Los Angeles, California 90095

## Abstract

The ability of the central nervous system to synthesize steroid hormones has wide-ranging implications for physiology and pathology. Among the proposed roles of neurosteroids is the regulation of the LH surge. This involvement in the estrogen-positive feedback demonstrates the integration of peripheral steroids with neurosteroids. Within the female hypothalamus, estradiol from developing follicles stimulates progesterone synthesis in astrocytes, which activate neural circuits regulating gonadotropin (GnRH) neurons. Estradiol acts at membrane estrogen receptor-α to activate cellular signaling that results in the release of inositol trisphosphate-sensitive calcium stores that are sufficient to induce neuroprogesterone synthesis. The purpose of the present studies was to characterize the estradiol-induced signaling leading to activation of steroid acute regulatory protein (StAR) and transporter protein (TSPO), which mediate the rate-limiting step in steroidogenesis, ie, the transport of cholesterol into the mitochondrion. Treatment of primary cultures of adult female rat hypothalamic astrocytes with estradiol induced a cascade of phosphorylation that resulted in the activation of a calcium-dependent adenylyl cyclase, AC1, elevation of cAMP, and activation of both StAR and TSPO. Blocking protein kinase A activation with H-89 abrogated the estradiol-induced neuroprogesterone synthesis. Thus, together with previous results, these experiments completed the characterization of how estradiol action at the membrane leads to the augmentation of neuroprogesterone synthesis through increasing cAMP, activation of protein kinase A, and the phosphorylation of TSPO and StAR in hypothalamic astrocytes.

Estradiol (E_2_) signaling plays a central role in the regulation of the hypothalamo-pituitary-ovarian axis. During most of the estrous cycle, E_2_ negatively feeds back onto the hypothalamus, preventing the surge release of GnRH. On proestrus, however, rising levels of E_2_ indirectly and positively signal the release of GnRH, stimulating the LH surge and subsequent ovulation (1). The rising levels of E_2_ also stimulate neuroprogesterone synthesis in the hypothalamus, a critical step modulating estrogen positive feedback and ovulation (2, 3). Blocking steroidogenesis in the hypothalamus of E_2_-treated ovariectomized and adrenalectomized rats or in gonadally intact rats prevents the LH surge (2, 4–6). Astrocytes are the most steroidogenic cells in the nervous system, and E_2_ stimulates neuroprogesterone synthesis in hypothalamic astrocytes (7–9). The E_2_ facilitation of neuroprogesterone synthesis requires membrane estrogen receptor-α (ERα) and the transactivation of metabotropic glutamate receptor-1a (mGluR1a), which induces a robust elevation of intracellular free calcium ([Ca^2+^]_i_) through the phospholipase C generation of inositol trisphosphate (IP_3_) (10, 11). The release of calcium (Ca^2+^) is necessary for neuroprogesterone synthesis (Figure 1) (10, 12, 13), but preliminary results indicated that E_2_ treatment of astrocytes induces protein kinase A (PKA). The present experiments were done to characterize the pathway through which E_2_ action at the membrane modulates the rate-limiting step of steroidogenesis, that is, the transport of cholesterol to the inner mitochondrial membrane. In classical steroidogenic tissue (eg, ovary and adrenal) as well as astrocytes, translocator protein (TSPO; also known as peripheral-type benzodiazepine receptor) appears to interact with steroid acute regulatory protein (StAR) to mediate this cholesterol transport function (14, 15) (but see Ref. 16).

**Figure 1.**
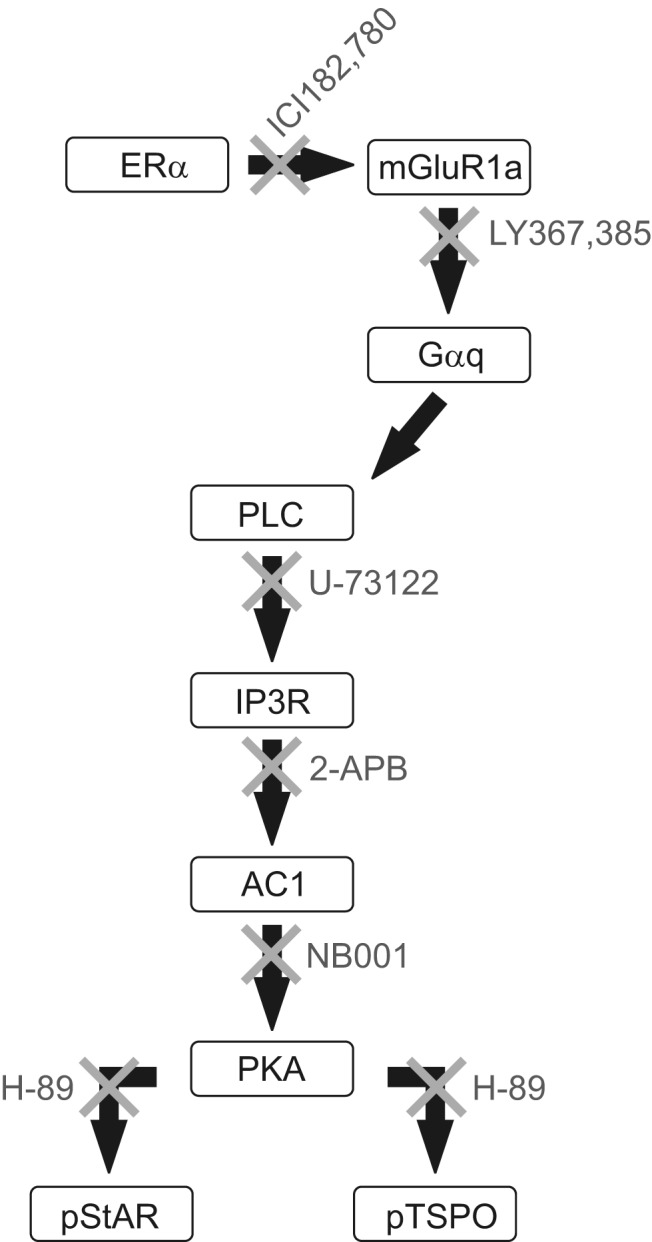
A schematic of the pathway leading to the activation of TSPO in astrocytes. Estradiol activates ERα, transactivating mGluR1a and stimulating the release of internal stores of Ca^2+^ from IP_3_ receptor-sensitive stores. This [Ca^2+^]_i_ activates AC1, generating cAMP to stimulate PKA, phosphorylating StAR and TSPO, and facilitating the import of cholesterol into the mitochondrion. Here, CYP11A1 cleaves the side chain producing pregnenolone, which is converted to progesterone by 3β-hydroxysteroid dehydrogenase either in the mitochondrion or the smooth endoplasmic reticulum. The inhibitors are listed below each step.

In vivo, a microarray of phosphorylated proteins indicated that E_2_ treatment activated both PKC and PKA in hypothalamic tissue (17). Although both kinases have been reported to increase the activity of TSPO and StAR initiating steroid biosynthesis, it is not clear which pathway is involved in astrocytes (18–20). The Ca^2+^ dependence of neuroprogesterone synthesis suggests that a PKC mechanism is principally involved in this regulation. However, PKA has been shown to be the primary modulator of StAR and TSPO activity (21–23). Thus, in the present experiments, we need to understand the role of E_2_-induced [Ca^2+^]_i_ in the regulation of PKC or PKA activation and neuroprogesterone synthesis.

Using primary cultures of postpubertal female hypothalamic astrocytes, we determined that E_2_ stimulated phosphorylation of StAR and TSPO by PKA and that blocking PKA activation abrogated the synthesis of neuroprogesterone. Estradiol treatment activated PKA through a Ca^2+^-dependent adenylyl cyclase-1 (AC1) (24, 25), which increased levels of cAMP. Antagonizing IP_3_ receptors prevented the E_2_-induced phosphorylation of PKA. Interestingly, we did not detect an E_2_ induction of PKCθ in astrocytes, whereas we had measured such an increase in neurons (17, 26, 27). Antagonizing PKA blocked the E_2_-induced neuroprogesterone synthesis while also preventing the activation of TSPO and StAR. Together with previous results from our laboratory, the present results indicate that the E_2_ control of the rate-limiting step in neuroprogesterone synthesis in astrocytes requires induction of a pathway from membrane ERα to IP_3_ receptor-dependent Ca^2+^ release and stimulation of AC1. The increased intracellular level of cAMP increased PKA phosphorylation and activated StAR and TSPO to facilitate cholesterol transport to the mitochondrial inner membrane increasing steroidogenesis (Figure 1). Some of these results have been presented earlier in preliminary form (28).

## Materials and Methods

All experimental procedures were performed in accordance with the principles and procedures of the National Institutes of Health Guide for the Care and Use of Laboratory Animals and approved by the Chancellor's Animal Research Committee at the University of California at Los Angeles.

### Primary cell culture

Primary hypothalamic astrocyte cultures were prepared from 4 to 6 Long-Evans female rats (40–50 days old) (Charles River) as described previously (12, 13, 29). Briefly, hypothalamic blocks were dissected with the following boundaries: rostral extent of the optic chiasm, rostral extent of the mammillary bodies, lateral edges of the tuber cinereum, and the top of the third ventricle. The tissue was minced, dissociated by mechanical and enzymatic treatment with 2.5% trypsin solution (Invitrogen), and plated in T-75 flasks. Cultures were maintained at 37°C with 5% CO_2_ and grown in DMEM/F12 (Mediatech), supplemented with 20% fetal bovine serum (FBS) (Invitrogen), 1% penicillin (10 000 IU/mL)/streptomycin (10 000 μg/mL) solution (Mediatech), and 2.5mM l-glutamine (Hyclone) for 3 days before the medium was replaced. After 12 days, FBS was decreased to 10%. Astrocyte cultures were grown to confluence and shaken on an orbital shaker at 200 rpm at 37°C for 18 to 24 hours to eliminate oligodendrocytes and microglia (30). For quantitative real-time PCR (RT-PCR), 200 000 cells per well were replated into 6-well plates.

Before experimental manipulations, astrocytes were steroid-starved for 18 to 24 hours with 5% charcoal-stripped FBS in DMEM/F12 medium. Cultures were then treated with vehicle dimethylsulfoxide or 17β-estradiol (10^−9^M–10^−7^M; Sigma-Aldrich) in steroid-free media (see above) for 20 minutes, 24 hours, or 48 hours at 37°C. Cells were collected for immunoblotting and quantitative RT-PCR after E_2_ treatment.

### Quantitative RT-PCR analysis

To determine the E_2_ regulation of TSPO, we initially tested whether E_2_ increased the levels of *TSPO* mRNA. The mRNA levels were measured in postputbertal female rat hypothalamic astrocytes by quantitative RT-PCR. Briefly, total RNA was isolated from primary astrocyte cultures using TRIZOL reagent (Invitrogen) according to the manufacturer's protocol. Total RNA was quantified using a NanoDrop ND-1000 spectrophotometer (Thermo Scientific). cDNA was synthesized from the total cellular RNA using random hexamers and Superscript III RNase H reverse transcriptase (Invitrogen). Quantitative real-time PCRs were performed in the Mx3000P real-time PCR system (Agilent) using TaqMan gene expression assays (Applied Biosystems) for *TSPO* (assay ID RN00560892_m1). The primers in this gene expression assay amplified a 117-bp region spanning exons 3 and 4 (accession no. NM_012515.2). The PCR conditions used for amplification were an initial setup for 2 minutes at 50°C and 10 minutes at 95°C, denaturation for 15 seconds at 95°C, and annealing for 1 minute at 60°C for 60 cycles. Samples were run in duplicate. Negative controls, without reverse transcriptase, and water controls were included in each reaction. Relative gene expression data were analyzed using the comparative threshold cycle (Ct) method (31). The Ct values of *TSPO* mRNA level were normalized to the endogenous gene *GAPDH* mRNA level present within each sample well.

### Immunoblotting

For Western blotting, astrocyte cultures (1 T-75 flask per treatment) were washed 3 times with HEPES buffer and collected by mechanical scraping (Corning). The samples were centrifuged (1800 rpm for 5 minutes), and the cell pellets were lysed in Lysis M buffer (Roche) with protease inhibitor and phosphatase inhibitor (Invitrogen). Protein concentrations were determined using the bicinchoninic acid method. Protein (30 μg) of each sample was loaded on a 10% SDS or Any KD polyacrylamide gel (Bio-Rad) and separated by electrophoresis. Proteins were transferred onto polyvinylidene fluoride membranes through a semidry transfer procedure (Bio-Rad). For detection of phospho-proteins, phosphoprotein-binding resin columns (Thermo Fisher Scientific) were used when antibodies were not available or effective. Membranes were probed with primary rabbit polyclonal antibodies: TSPO (1:1000; Trevigen, Inc), StAR (1:1000; Abcam), phospho-PKA (Thr197) (1:500; Cell Signaling), phospho-PKC (Thr538) (1:500; Cell Signaling), PKA (1:1000; Cell Signaling), PKC (1:1000; Cell Signaling), and AC1 (ab38331; Abcam). All antibodies were diluted in 5% nonfat dry milk or 5% bovine serum albumin in Tris-buffered saline/Tween 20 (0.1% [vol/vol]) according to the suppliers' recommendations. Blots were then incubated with goat antirabbit IgG horseradish peroxidase (1:10,000; Santa Cruz Biotechnology, Dallas, TX), or goat antirabbit IgG (H+L) (1:10,000; Vector Laboratories) for 1 hour at room temperature. Bands were visualized using an enhanced chemiluminescence (ECL) kit (Thermo Fisher Scientific). β-Actin (1:1000; Cell Signaling) was used as a loading control, and immunoreactive bands were normalized to obtain the percentage of protein to β-actin ratio. Bands were immediately visualized using FluorChem E and analyzed by Alphaview software (Cell Bioscience).

### cAMP ELISA

Cells were replated onto 6-well plates and steroid-starved for 18 hours as indicated above. After steroid starvation, cells were incubated with 250mM 3-isobutyl-1-methylxanthine (Sigma) for 30 minutes to inhibit cAMP-metabolizing phosphodiesterases. Cells were pretreated with vehicle or 100μM NB001 (AC1 inhibitor; Sigma-Aldrich) for 15 minutes before the 20-minute vehicle or 1nM E2 treatment in steroid-free media. Cells were washed once with ice-cold Hank's Balanced Salt Solution and lysed in 0.1N HCl, 0.5% Triton X-100. Lysates were clarified with centrifugation at 10 000*g* for 5 minutes. Intracellular cAMP levels were quantified from the supernatants using the cAMP complete ELISA kit (Enzo Life Sciences) according to the manufacturer's instructions using the acetylation protocol. Interassay coefficients of variation were between 4.6% and 8.4%, and intra-assay coefficients of variation were between 7.8% and 13.6% for the acetylated version of the assay according to the manufacturer. ELISA data were normalized to protein concentrations of the lysates.

### Progesterone RIA

Astrocytes (500 000) were plated into 6-well culture plates and grown for 24 hours. Astrocytes were then steroid-starved for 18 hours before experimentation. After the 18-hour steroid starvation, culture medium was removed, 1 mL of H-89 (100μM) was added to each well and incubated for 5 minutes, the supernatant was collected, and the samples were frozen at −4°C. For RIA, samples were thawed, mixed with diethyl ether (Fisher Scientific), vortexed for 2 minutes, and placed into a methanol and dry ice bath to freeze the aqueous layer. The upper ether layer was decanted into a separate tube, and the ether was allowed to evaporate overnight. The extract was reconstituted in isooctane (Mallinkrodt Baker) and a Celite column (Sigma-Aldrich) with ethylene glycol as the stationary phase used to isolate the progesterone. Progesterone was then eluted off the column using 4 mL of isooctane. Standards and samples (100 μL) were incubated with rabbit polyclonal antibody against progesterone (Sigma-Aldrich) for 30 minutes at 37°C. Tritium-radiolabeled progesterone (2000 cpm/mL) was then added and incubated for an additional 60 minutes at 37°C. Standards and samples were cooled at 4°C, and a 0.05% charcoal dextran solution (Sigma-Aldrich) was added to remove all unbound progesterone. The mixture was centrifuged (3000 rpm) for 15 minutes at 4°C. All samples were run in duplicate, and sample progesterone concentrations determined by extrapolation from a curve determined from the progesterone standards. The intra- and interassay coefficients of variation for progesterone were 3.2% and 4.8%, respectively.

### Statistical analysis

Data are presented as means ± SEM of a percent relative ratio. Statistical comparisons were made using unpaired Student's *t* test when comparing means between 2 independent groups, and one-way ANOVA with Student-Newman-Keuls (SNK) post hoc test when comparing means across 3 or more independent groups. Values of *P* < .05 were considered significant. For RT-PCR, the relative difference between sample groups was calculated according to the difference in Ct values using 2^−Δ(Δ Ct)^ (31).

## Results

**Figure 2.**
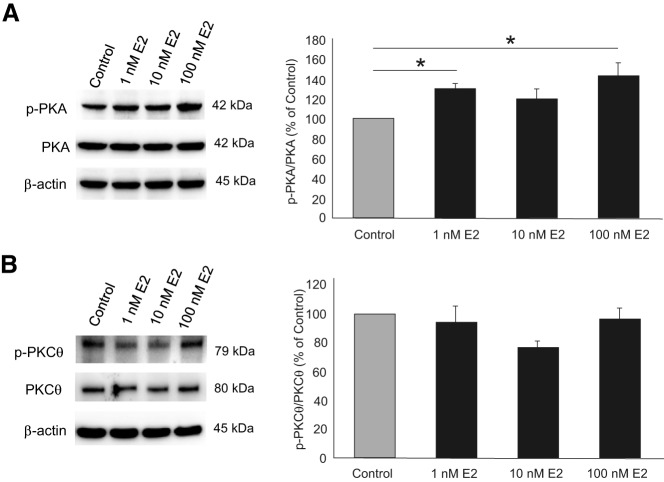
Effect of E_2_ on phosphorylation of PKA in primary cultures of astrocytes obtained from postpubertal female rats. All doses of E_2_ activated PKA, but not PKCθ. Vehicle control (no E_2_) was set as 100%. The other cultures were treated with E_2_ for 20 minutes. In B, values represent means (n = 5 independent experiments) ± SEM of relative ratio normalized to the vehicle control (no E_2_) that was set as 100%. *, Significant difference from controls (*P* < .05) as determined by one-way ANOVA with SNK post hoc test.

In primary hypothalamic astrocyte cultures derived from postpubertal female rats, E_2_ treatment induced the PKA pathway (Figure 1). A 20-minute treatment with either 1nM or 100nM E_2_ increased PKA phosphorylation levels by 30% to 40% (Figure 2A; 1nM E_2_: 130.59% ± 5.01%, 100nM E_2_: 143.72% ± 13.23% vs control; one-way ANOVA; *df* = 3, 36; *F* = 4.161; SNK *P* < .05). Because E_2_ has been demonstrated to activate PKA and PKCθ in the hypothalamus in vivo (16), we also measured the E_2_ induction of phospho-PKCθ in vitro (Figure 2). Treatment with 1nM, 10nM, or 100nM E_2_ did not increase levels of phosphorylated PKCθ (Figure 2B; 94.17% ± 11.34% vs control; one-way ANOVA; *df* = 3, 19; *F* = 2.041; SNK *P* > .05).

**Figure 3.**
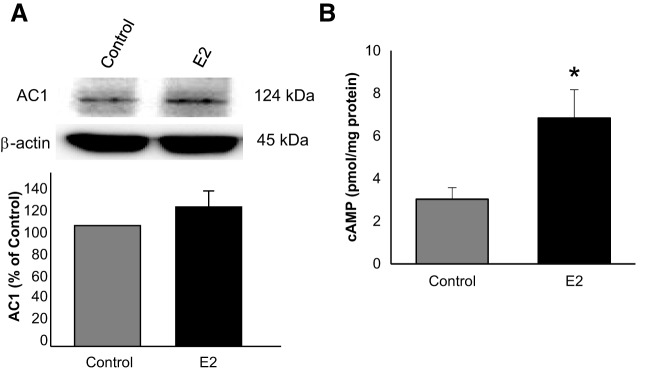
E_2_ treatment increased cAMP in primary astrocyte cultures. A, Whole-cell lysates were then separated and identified with Western blotting. E_2_ (1nM) did not induce Ca^2+^-dependent AC1 activity. B, E_2_ (1nM) increased intracellular cAMP levels more than 2-fold as determined by a competitive ELISA for cAMP. Values represent means ± SEM. *, *P* < .05, as determined by unpaired Student's *t* test of 4 independent experiments.

**Figure 4.**
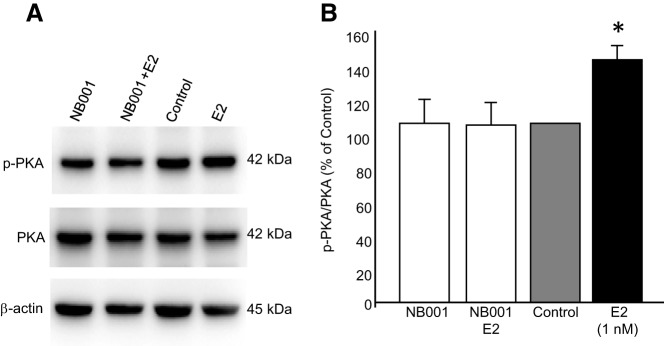
Inhibition of AC1 abolished PKA phosphorylation. Cultures of postpubertal hypothalamic astrocytes were pretreated for 15 minutes with NB001 (an AC1 inhibitor) or vehicle and then treated with 1nM E_2_ or vehicle for 20 minutes. Stimulation with E_2_ alone induced PKA phosphorylation, whereas treatment with NB001 plus E_2_ prevented PKA activation. Values are means ± SEM of relative ratio normalized to the vehicle control (no NB001, no E_2_) set as 100%. *, *P* < .05, as determined by one-way ANOVA with SNK post hoc test.

PKA is activated by cAMP generated by adenylyl cyclase. Because a hallmark of E_2_ action in astrocytes is the elevation of [Ca^2+^]_i_, we investigated whether E_2_ induced the Ca^2+^-dependent AC1. Primary astrocyte cultures were stimulated with E_2_ for 20 minutes, harvested, and processed for Western blotting. Estradiol treatment did not significantly increase AC1 levels (Figure 3; 121.6% ± 9.7% vs control; unpaired *t* test *df* = 6; *P* = .0657, *t* = −4.671), but cAMP levels were significantly increased (Figure 3B; E_2_: 6.53 ± 1.07 pmol cAMP/mg protein vs control: 2.78 ± 0.50 pmol cAMP/mg protein; unpaired *t* test *df* = 8; *P* = .013, *t* = −3.173). To demonstrate that E_2_-induced activation of PKA depended on AC1, astrocyte cultures were incubated with NB001, an AC1 inhibitor (100μM; Sigma-Aldrich), or vehicle (dimethylsulfoxide) before E_2_ treatment. E_2_ exposure significantly elevated phospho-PKA levels, and this increase was abrogated by NB001 (Figure 4; E_2_: 141.12% ± 3.96% vs NB001 plus E_2_: 94.78% ± 8.55%; one-way ANOVA *df* = 3, 12; *F* = 11.732; SNK *P* < .01).

**Figure 5.**
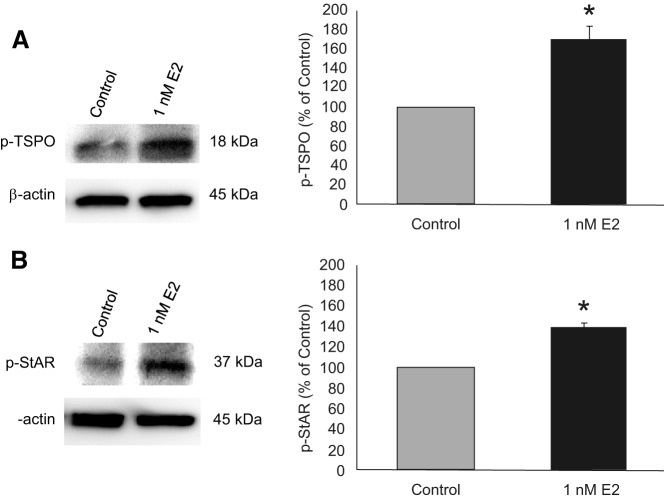
The effect of E_2_ on phosphorylation of TSPO and StAR in hypothalamic astrocytes. Astrocyte cultures were incubated with 1nM E_2_ for 20 minutes. After E_2_ treatment, phosphorylated proteins were isolated from whole-cell lysates using a phosphoprotein-binding resin column and analyzed by immunoblotting. Phospho-TSPO and phospho-StAR were normalized to β-actin. A, E_2_ treatment increased TSPO phosphorylation by 70% (n = 5 independent experiments). B, E_2_ treatment increased StAR phosphorylation by 40% (n = 4 independent experiments); cytoplasmic, active phospho-StAR was observed at 37 kDa. Vehicle control (no E_2_) was set as 100%. Values are means ± SEM. *, *P* < .05, as determined by unpaired Student's *t* test.

E_2_ treatments for various times (20 minutes, 24 hours, or 48 hours) did not increase *TSPO* mRNA levels (one-way ANOVA *df* = 3, 15; *F* = 1.563; SNK *P* > .05; data not shown). These results indicate that TSPO was not regulated through a transcriptional mechanism. To examine whether TSPO was phosphorylated in response to E_2_ treatment, astrocyte cultures were harvested, and whole-cell lysates passed through a phosphoprotein-binding resin column. Retained phosphorylated proteins were eluted and analyzed by Western blotting using a TSPO-specific antibody. At 20 minutes, 1nM E_2_ treatment produced a 70% increase in intracellular levels of phosphorylated TSPO (Figure 5A; 170.18% ± 12.65% vs control; unpaired *t* test *df* = 8; *P* = .0005, *t* = −5.550). Because *StAR* mRNA is not increased by E_2_ treatment in vitro (32), we examined whether phosphorylation of StAR was regulated by membrane-initiated E_2_ signaling. Like TSPO, 1nM E_2_ treatment increased levels of phosphorylated StAR (Figure 5B; 138.55% ± 4.28% vs control; unpaired *t* test *df* = 8; *P* < .0001, *t* = 9.009).

**Figure 6.**
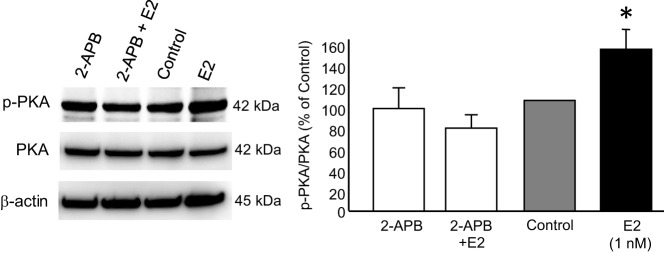
Inhibition of IP_3_ receptor abolished PKA phosphorylation. Postpubertal hypothalamic astrocytes were incubated with IP_3_ receptor inhibitor 2-APB before E_2_ stimulation, and the effect of 2-APB on PKA phosphorylation were determined. 2-APB significantly blocked the E_2_-induced cAMP-PKA signaling (n = 4 independent experiments). Values are means ± SEM of relative ratio normalized to the vehicle control (no 2-APB, no E_2_) set as 100%. *, *P* < .05, as determined by one-way ANOVA with SNK post hoc test.

E_2_ induces a robust increase in [Ca^2+^]_i_ in primary astrocytes, and this increase is necessary for neuroprogesterone synthesis (10, 11). To test the role of activating IP_3_ receptors in PKA activation, astrocytes were treated with the IP_3_ receptor inhibitor 2-aminoethoxydiphenyl borate (2-APB; 5μM), 5 minutes before E_2_ stimulation. 2-APB inhibited E_2_-induced PKA phosphorylation (Figure 6; E_2_: 137.97% ± 14.75% vs 2-APB plus E_2_: 86.91% ± 6.43%; one-way ANOVA *df* = 3, 12; *F* = 7.909; SNK *P* = .0036).

**Figure 7.**
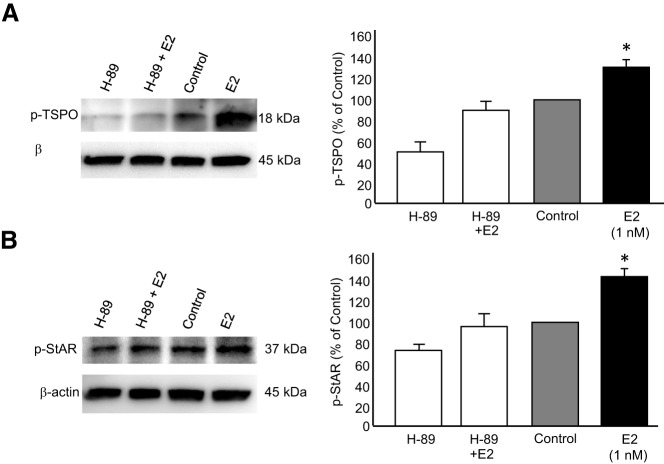
Inhibition of PKA prevented TSPO and StAR phosphorylation. Postpubertal hypothalamic astrocyte cultures were pretreated with PKA inhibitor H-89, followed by a 20-minute E_2_ treatment. A, H-89 significantly prevented TSPO phosphorylation (n = 5 independent experiments). B, H-89 significantly inhibited StAR phosphorylation (n = 4 independent experiments). Values are means ± SEM of relative ratio normalized to the vehicle control (no H-89, no E_2_) set as 100%. *, *P* < .05, as determined by one-way ANOVA with SNK post hoc test.

To determine whether E_2_-induced PKA-regulated TSPO and StAR activity, hypothalamic astrocyte cultures were treated with a PKA inhibitor, *N-*[2-[[3-(4-bromophenyl)-2-propenyl-]amino]ethyl]-5-isoquinoline-sulfonamide dihydrochloride (H-89; 25μM). A 30-minute pretreatment with H-89 prevented E_2_ induction of TSPO phosphorylation (Figure 7A; E_2_: 148.07% ± 4.98% of control vs H-89 plus E_2_: 85.93% ± 7.39%; one-way ANOVA *df* = 3, 16; *F* = 60.366; SNK *P* < .0001). Similarly, H-89 inhibited StAR phosphorylation (Figure 7B; E_2_: 138.55% ± 4.28% vs H-89 plus E_2_: 91.18% ± 9.56%; one-way ANOVA *df* = 3, 14; *F* = 29.419; SNK *P* < .0001). Together these data suggest that a PKA-mediated mechanism activated TSPO and StAR, inducing cholesterol transport.

**Figure 8.**
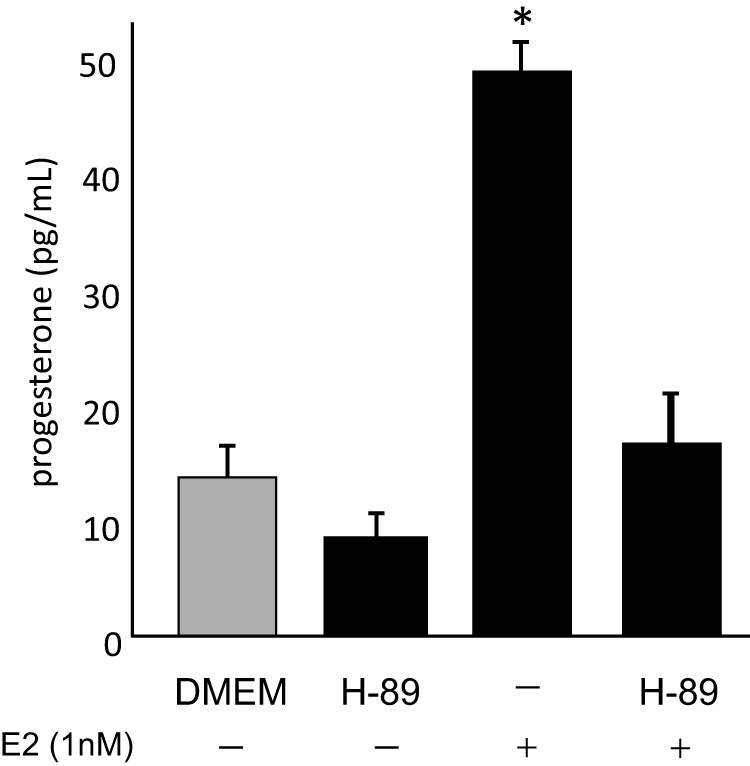
Effect of H-89, a PKA inhibitor, on E2-induced neuroprogesterone synthesis in postpubertal astrocytes in vitro. Astrocytes had a robust facilitation of neuroprogesterone synthesis upon exposure to E_2_ (1nM; *P* < .05 vs DMEM control). H-89 by itself did not significantly reduce basal neuroprogesterone synthesis in female astrocytes (*P* > .05 vs control). Treatment of astrocytes with H-89 prevented the E_2_-stimulated neuroprogesterone synthesis (*P* > .05 vs control). Values are means of 6 samples ± SEM. *, Significant difference (*P* < .05 compared with control [DMEM] determined by one-way ANOVA with SNK post hoc test).

To ascertain whether blocking PKA disrupted estradiol-induced progesterone synthesis in astrocytes, postpubertal astrocyte enriched cultures were treated with a PKA inhibitor, H-89, in the presence or absence of E_2_. H-89 blocked estradiol-induced progesterone synthesis (Figure 8; *df* = 3,12; *F* = 13.2; *P* < .001).

## Discussion

Previous studies have demonstrated that the E_2_-induced increase of neuroprogesterone synthesis in hypothalamic astrocytes is rapid and does not appear to be dependent on de novo gene transcription (12, 32). Several observations are consistent with this idea, including E_2_ membrane-induced signaling and the release of intracellular stores of Ca^2+^. Release of Ca^2+^ without E_2_ stimulation was sufficient to increase neuroprogesterone synthesis in astrocytes (32) and suggested that this facilitation required an increased transport of cholesterol into the mitochondrion. This is the rate-limiting step of steroidogenesis and is mediated by the transport proteins TSPO and StAR. Thus, the major findings of these studies are that the E_2_-induced synthesis of neuroprogesterone in female hypothalamic astrocytes is regulated by the PKA pathway, probably through activation of StAR and TSPO. StAR is a hormone-induced protein that is targeted to the mitochondrion and is critical for TSPO-mediated intracellular lipid transport into the mitochondrial matrix (21, 33). Here, along the inner mitochondrial membrane, cholesterol is converted to pregnenolone by cytochrome P450 side-chain cleavage enzyme (CYP11A1), the first enzymatic step of steroid biosynthesis. The present results bring together disparate observations about E_2_ action in astrocytes and the synthesis of neuroprogesterone. First, E_2_-induced release of IP_3_-sensitive stores of Ca^2+^, which we showed was necessary for the generation of cAMP needed for PKA phosphorylation. Second, E_2_ activation of PKA was necessary for phosphorylation of StAR and TSPO. Blocking PKA prevented E_2_-induced neuroprogesterone synthesis. This is similar to the hormone-stimulated phosphorylation of StAR and TSPO in various steroidogenic cells that has been has been well studied (34, 35). But to our knowledge, this is the first report in astrocytes to demonstrate the pathway through which E_2_ membrane-initiated signaling led to activation of StAR and TSPO and a rapid facilitation of neuroprogesterone synthesis.

In vivo, E_2_-induced neuroprogesterone synthesis is critical for stimulating the LH surge and ovulation in rodents (reviewed in Ref. 36). Blocking steroidogenesis in rodent hypothalamus prevents estrogen positive feedback and ovulation (2, 5). Moreover, these results are consistent with the action of mifepristone in women taking oral contraceptives and suggest that extraovarian progesterone synthesis is required for the LH surge in humans (37). Although astrocytes from other regions are capable of synthesizing progesterone, only postpubertal astrocytes derived from the female hypothalamus are capable of responding to E_2_ with elevated synthesis (2, 13). Consistent with this observation, male rodents do not exhibit an estrogen positive feedback-induced LH surge (2, 38). As with estrogen positive feedback, the E_2_ facilitation of neuroprogesterone synthesis in hypothalamic astrocytes is sexually differentiated through gonadal steroid action during development (38).

Treatment of female primary adult astrocytes with E_2_, in vitro, rapidly elicits a dramatic increase in [Ca^2+^]_i_, which facilitates neuroprogesterone synthesis (32). This release of intracellular stores of Ca^2+^ is mediated by membrane ERα transactivating the mGluR1a and the generation of IP_3_ (12). It had not been clear how this fit with the observed E_2_ regulation of levels of TSPO ligand binding and *TSPO* mRNA (39–42). Moreover, an analysis of the amino acid sequence of the rat *TSPO* gene revealed the presence of several putative phosphorylation sites (43). In peripheral tissues, PKA phosphorylates TSPO, and this phosphorylation is dependent on cAMP (34). The present experiments defined a pathway from membrane-initiated E_2_ release of Ca^2+^ to increased cAMP and the activation of PKA. The key findings are that blocking AC1 or PKA activation prevented TSPO and StAR activation, and blocking PKA prevents E_2_-induced neuroprogesterone synthesis, which is consistent with the report of PKA-mediated TSPO phosphorylation (33).

We observed differences between the effects of E_2_ treatment of primary cultures of postpubertal astrocytes compared with the in situ hypothalamic response to E_2_. In vivo, E_2_ induces the transcription of 3β-hydroxysteroid dehydrogenase (*HSD3B1*), an enzyme critical to steroidogenesis, but not *StAR* or *CYP11A1* (44). In astrocyte cultures, however, we did not detect any change in the mRNA levels of steroidogenesis-associated proteins (32). Similarly, in the present study, TSPO mRNA was not increased. Although E_2_ may regulate TSPO transcription through an estrogen response element, the acute increase of steroidogenesis in astrocytes requires a different mechanism (32, 42). By demonstrating that E_2_ induced PKA, the present experiments uncover the mechanism by which E_2_ membrane-initiated signaling facilitates neuroprogesterone synthesis (Figure 1). Before these experiments, and based on the E_2_-induced increase in [Ca^2+^]_i_, we assumed that stimulation of neuroprogesterone synthesis in astrocytes was associated with PKC. In neurons, E_2_ induces PKCθ phosphorylation, an action that activates sexual receptivity (17). In distinction to neurons, astrocytes had an E_2_-induced increase in [Ca^2+^]_i_, stimulating a Ca^2+^-sensitive AC1 and resulting in elevated cAMP levels and activating PKA.

StAR appears critical for the acute regulation of steroidogenesis along with TSPO (45). The amino acid sequence of StAR contains 2 consensus sequences for PKA phosphorylation sites. In particular, a phosphorylation site at serine 194/195 mediated by PKA allows StAR to become fully active, allowing its insertion into the mitochondrial membrane (46). The present results are consistent with the proposed mechanism of hormonally induced steroidogenesis, which needs very rapid phosphorylation and activation of StAR and TSPO, to facilitate the transport of cholesterol to the inner mitochondrial membrane where CYP11A1 cleaves the cholesterol side chain forming pregnenolone (47). The interaction of StAR and TSPO in the mitochondrial membrane was revealed by fluorescent resonance energy transfer analysis (48). In MA-10 Leydig cells, hormone stimulation induced the colocalization of TSPO, PKA regulatory subunit RIα-associated protein 7 (PAP7), and StAR in mitochondria (21). Inhibition of StAR and TSPO abrogate the hormonally induced progesterone synthesis (49). In astrocytes, the described PKA-mediated mechanism allows for the rapid facilitation of neuroprogesterone synthesis, which can be measured within minutes of E_2_ treatment (12). The described phosphorylation cascade fits the time course of this rapid activation of already existing proteins leading to phosphorylation of StAR and TSPO.

The 37-kDa precursor of StAR is a cytoplasmic protein and has an NH_2_-terminal mitochondrial-targeting sequence. Phosphorylation activates StAR and facilitates cholesterol transport. The importation of the 37-kDa StAR into the mitochondrion results in cleavage to a 32-kDa intermediate and eventually to the mature, inactive 30-kDa intramitochondrial protein (47, 49–53). Upon hormonal stimulation, StAR-expressing cells convert cholesterol to pregnenolone, suggesting that StAR is involved in rapid steroidogenesis (54). We and several others have identified StAR in the brain and in particular in astrocytes (32, 44, 55–57). cAMP upregulated StAR expression in mesencephalic and cerebellar astrocytes, which increased pregnenolone synthesis after 12 to 24 hours (58), but in our experiments, the time course was much more rapid, 5 to 60 minutes (13, 32).

TSPO was originally discovered as a binding site for the benzodiazepines in peripheral tissue, hence its original name, peripheral benzodiazepine receptor (PBR) (59, 60). The cholesterol recognition amino acid consensus (CRAC) domain of TSPO appears to be responsible for the mitochondrial uptake and translocation of cholesterol (61, 62). Not surprisingly, the most studied role of TSPO is in steroidogenesis (15). Agonists of TSPO stimulate steroidogenesis in both peripheral tissues and in the brain, and knockdown of TSPO abrogates steroidogenesis (63, 64). However, TSPO functionality requires the interaction with several mitochondrial membrane proteins that form a 140- to 220-kDa complex consisting of the voltage-dependent anion channel (VDAC1), adenine nucleotide transporter (ANT), diazepam-binding inhibitor (acyl-coenzyme A-binding domain 1, ACBD1), the TSPO-associated protein-1 (PRAX-1), and the PKA regulatory subunit RIα-associated protein 7 (PAP7) (45, 65). This multiprotein complex is downstream of, and regulated by, StAR (21, 49), which can be demonstrated by the highly attenuated, but still extant, steroidogenesis in the absence of StAR (66, 67).

Although our laboratory's focus is on the regulation of reproduction, and particularly on central nervous system control of the LH surge, the present findings have salience to a wide range of actions in which neurosteroids and TSPO have been implicated, including apoptosis, differentiation, neuroprotection (encompassing neurodegenerative, ischemic, and traumatic insults), cognition, and anxiolysis (42). The role of steroidogenesis in the nervous system has been reported to be both deleterious and beneficial. Recently, TSPO has been implicated in several central nervous system pathologies. In a model of multiple sclerosis, TSPO is upregulated in microglia and astrocytes during neural inflammation. Significantly, TSPO downregulation has been shown to be beneficial for MS (68). However, in rat models of axotomy and neuropathy, TSPO ligands improved neural survival and repair, suggesting that TSPO-mediated steroidogenesis could be a potential treatment for neurodegenerative diseases (69). In the 3xTgAD mouse model of Alzheimer's disease, TSPO activation was shown to enhance steroidogenesis and neuroprotection (70). Moreover, activation of TSPO has been shown to have anxiolytic actions potentially due to an increase in synthesis of neurosteroids with sedative properties (64, 71, 72). Patients with posttraumatic stress disorder have lower levels of TSPO, and stimulation of TSPO improved behavioral deficits in a mouse model of posttraumatic stress disorder (73–75).

In summary, the present results have mapped the pathway of E_2_-induced facilitation of neuroprogesterone synthesis, which involves the release of intracellular Ca^2+^ stores, elevating cAMP levels, activating PKA and the phosphorylation of both StAR and TSPO, stimulating the transport of cholesterol into the mitochondrial matrix, and increasing the synthesis of progesterone.
